# Pretreatment Neutrophil to Lymphocyte Ratio (NLR) Predicts Prognosis for Castration Resistant Prostate Cancer Patients Underwent Enzalutamide

**DOI:** 10.1155/2019/9450838

**Published:** 2019-01-20

**Authors:** Yohei Kumano, Yoriko Hasegawa, Takashi Kawahara, Masato Yasui, Yasuhide Miyoshi, Nobuaki Matsubara, Hiroji Uemura

**Affiliations:** ^1^Department of Urology and Renal Transportation, Yokohama City University Medical Center, Yokohama, Japan; ^2^Department of Breast and Medical Oncology, National Cancer Center Hospital East, Tokyo, Japan

## Abstract

**Introduction:**

Nearly 80% of advanced prostate cancer patients respond to initial androgen deprivation therapy (ADT). However, ADT does not prevent the progression of prostate cancer over the long term, and the disease eventually progresses to castration-resistant prostate cancer (CRPC). Prior to the development of enzalutamide (ENZ) and abiraterone acetate, docetaxel was the only established treatment with life-prolongation for CRPC. ENZ is a second-generation anti-androgen receptor drug that has contributed to improving the prognosis of CRPC. Several studies have reported factors predicting the efficacy of ENZ; however, there are no confirmed biomarkers. The neutrophil-to-lymphocyte ratio (NLR) is an easily calculated biomarker that is associated with the prognosis of several solid malignancies. However, there were few studies investigated NLR for ENZ in patients with mCRPC. We examined the usefulness of the NLR as a predictive tool for ENZ.

**Methods:**

We retrospectively examined a total of 106 CRPC patients who were treated with ENZ until September 2016 in Yokohama City University Hospital, Yokohama City University Medical Center, and National Cancer Center Hospital East. ENZ was routinely started as a dose of 160 mg per day; the dosage was reduced in some patients due to side effects. Drug holiday for 1-2 weeks or dose reduction to 80-120mg was done and no patients discontinued ENZ treatment due to adverse effects. ENZ was stopped when cancer progression was detected based on PSA elevation, radiographic findings, and deterioration of the patient's performance status. The cut-off NLRs for overall survival (OS) and cancer-specific survival (CSS) were determined based on the receiver-operator curves. Kaplan-Meier curves were used to analyze the factors associated with OS or CSS and a log-rank test was performed. A multivariate analysis was also performed to analyze the factors associated with the prognosis.

**Results:**

We retrospectively reviewed 106 consecutive CRPC patients who were both treated with ENZ and were able to be counted before ENZ NLR. Cut-off point was 2.14 for both OS and CSS by receiver operator characteristic curve. The patients were then divided into the higher NLR group (≥2.14) and lower NLR group (<2.14). Multivariate analysis showed that NLR and predocetaxel chemotherapy were independent risk factors for both overall and cancer-specific survival.

**Conclusions:**

The NLR might be a useful biomarker for predicting the prognosis of mCRPC patients who are treated with ENZ.

## 1. Introduction

Prostate cancer is one of the most common malignancies and causes of cancer death among men worldwide [1]. Since the first report in 1941, androgen deprivation therapy has contributed to the management of almost every stage of prostate cancer [2–5]. Hormonal manipulation in men with prostate cancer can be achieved by reducing the availability of androgens and/or interference with their functions through the androgen receptor (AR) pathway. However, after a brief clinical response, most responders ultimately develop hormone-refractory tumors known as castration-resistant prostate cancer (CRPC). There is emerging evidence to support that AR activation is often associated with its overexpression in CRPC [6–9]. Previous reports suggested that hormone-refractory tumors still remain AR-dependent for their tumor growth. Enzalutamide (ENZ) is an oral AR signaling inhibitor that not only blocks androgen binding to AR-with a 5-8 fold higher affinity in comparison to bicalutamide, but also inhibits AR nuclear translocation, DNA binding, and coactivator recruitment [10]. The effectiveness of ENZ both before and after docetaxel chemotherapy was confirmed by the PREVEIL and AFFIRM trials [11]. Although some biomarkers, such as AR-V7 in circulating tumor cells, have been reported, there is a need for a more easily calculated biomarker for daily clinical use [12]. A recent study reported that the neutrophil-to-lymphocyte ratio (NLR) was an independent prognostic factor in some solid malignancies, including prostate cancer [13]. Our previous study also showed that the group with a higher NLR showed a poorer recurrence-free and overall survival in CRPC patients that underwent abiraterone acetate (ABI) comparing to the lower NLR group [13, 14]. Only two studies have examined the correlation between NLR and cancer progression for CRPC patients underwent ENZ [15, 16]. This study examined the potential application of the NLR as a biomarker in CRPC patients treated with ENZ.

## 2. Materials and Methods

### 2.1. Patients

We retrospectively reviewed 106 consecutive CRPC patients who were treated with ENZ from 2011 to 2016, at Yokohama City University Medical Center (Yokohama, Japan), Yokohama City University Graduate School of Medicine (Yokohama, Japan), and National Cancer Center Hospital East (Kashiwa, Japan). From 2011 to 2014, ENZ was administrated as a clinical trial courses. All patients were treated with standard dose and schedule of ENZ (160 mg, oral, once per day). This study was carried out in accordance with the ethical standards of the Declaration of Helsinki. The review boards of each institution approved this study (Yokohama City University Medical Center Institutional Review Board and National Cancer Center Hospital East Institutional Review Board).

### 2.2. Clinical Assessments

The primary endpoint of this study was assessing NLR as a prognostic factor for OS. OS was defined as survival from the start of ENZ treatment to death due to any cause. The secondary endpoint was the prostate-specific antigen (PSA) response. A PSA response was defined as a >50% reduction in the PSA level. ENZ was administered at a dose of 160 mg/day along with contentious surgical or medical castration (LH-RH agonists). After the initiation of ENZ treatment, the PSA level was measured every month, and ENZ treatment was continued until progression of the disease, as defined by the PCWG2 criteria, or until the development of unacceptable toxicity. Complete blood cell counts (CBCs) were performed, and the baseline NLR was calculated based on the neutrophil and lymphocyte counts obtained on the same day or a few days before the initiation of ENZ treatment. All data were retrospectively collected from clinical records.

### 2.3. Statistical Analyses

We used the area under the receiving operator characteristic (AUROC) curve to determine the ideal cut-off NLR. Median values were used as cut-off points for other factors (for continuous measurements). Multivariate logistic regression models were used to detect independent risk factors. A Kaplan-Meier product limit estimate was used to estimate the OS and CSS. The survival duration was defined as the time between the start of ENZ treatment and death of any cause. The log-rank test was performed for comparison between higher and lower NLR. P values of < 0.05 were considered to indicate statistical significance. The statistical analyses were performed using the SPSS (IBM, Chicago, IL, USA) and Graph Pad Prism (Graph Pad Software, La Jolla, CA, USA) software programs.

## 3. Results

The patients' clinical characteristics are shown in Table 1. The NLR was examined and median NLR of 3.8 (range: 0.5-23.1) before the start of ENZ treatment in a total of 106 patients. Based on ROC curve, the cut-off NLRs for the OS and CSS were both 2.14 (area under the ROC curve = 0.548, 0.584). We divided the patients into two groups based on the cut-off value: higher NLR group (≥2.14; n=71) and lower NLR group (<2.14; n=35).

**Figure 1:**
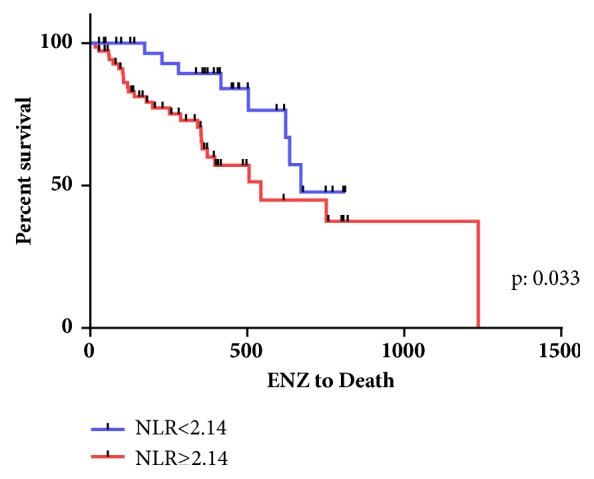
Kaplan-Meier curve for overall survival.

**Figure 2:**
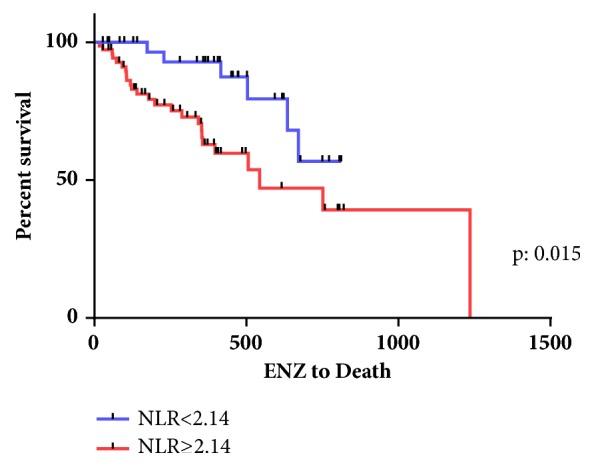
Kaplan-Meier curve for cancer specific survival.

The Kaplan-Meier curves for OS and CSS in the two groups are shown in Figures 1 and 2. The median OS and CSS in the higher NLR group were both 17.9 months, while those in the lower NLR group were 22.0 months and not yet reached. The log-rank test performed when creating the Kaplan-Meier curves for OS and CSS revealed that a higher NLR was associated with an increased risk of mortality in CRPC patients (HR: 2.27, 2.809 and p:0.015, 0.033, respectively).

A multivariate analysis of five factors (Gleason score, performance status, visceral metastasis, prior docetaxel chemotherapy, and NLR) was used to identify factors that were significantly associated with OS and CSS in CRPC patients. Two factors were identified as independent prognostic factors: higher-NLR (OS: HR=4.57; 95% CI 1.31-15.96; P=0.01, CSS: HR=8.26; 95% CI 1.59-42.76; P=0.01) and prior docetaxel chemotherapy (OS: HR=3.48; 95% CI 1.29-9.35; P=0.01, CSS: HR=4.8; 95% CI 1.54-14.98; P<0.01) (Tables 2 and 3).

## 4. Discussion

This study showed that higher NLR group showed poorer prognosis for CRPC patients underwernt ENZ. The NLR was first reported as an easy marker for predicting the general condition of cancer patients who were admitted to intensive care units due to major surgery, systemic inflammatory response syndrome, or sepsis [17]. The NLR can be easily calculated based on CBCs and does not require a special examination. The NLR might be a biomarker that predicts the response to ENZ in an era when there are no established biomarkers. Walsh et al. reported that the NLR was useful for predicting the prognosis of colorectal cancer patients [18, 19], and recent studies have reported that the NLR might be a useful predictive factor in prostate cancer [20, 21]. In ENZ, the assessment of NLR for CRPC patients who underwent NLR has been reported by Condeduca et al. and Choi et al. and higher NLR showed poorer prognostic factor for the patients treated by ENZ [15, 16].

Although some hypothesized mechanisms have been reported, the mechanism underlying the association between NLR elevation and cancer progression remains to be unestablished. Wenger et al. reported that the number of circulating lymphocytes was decreased by tumor enlargement, then resulting in higher NLR value [22]. Based on these findings, Gomez et al. suspected that elevation of NLR was represented about the condition of weak immunoreaction [18]. Recent studies revealed that some inflammatory cytokine marker like NLR also showed prognostic agent in some solid malignancies including CRPC [23–26]. Further study is needed about the correlation between systemic inflammation and cancer progression.

This study also showed that the NLR might be a useful predictor of CSS and OS in CRPC patients who are treated with ENZ. Our previous study showed that cut-off point of NLR was 3.76 for the CRPC patients receiving ABI [14] and our previous study also showed that NLR values were correlated with PSA value [13]. NLR value in this study was 2.14, based on these findings CPRC patients with NLR value less than 2.14 firstly tried ENZ might show favorable outcome

The present study is associated with several limitations. First, it was a retrospective study and some data related to the clinical course were missing. Secondly, the sample size was relatively small. To reveal the usefulness of the NLR as a biomarker, a longer term study of a larger population and prospective study should be performed. Finally, in Japan, tending to use multiple hormonal therapies comparatively on long term, the indications for ENZ treatment varied according to the physician. Thus, the NLR might reflect tumor aggressiveness rather than the efficacy of ENZ. Despite these limitations, the pre-ENZ NLR might be a predictor of cancer-specific and overall survival.

In conclusion, a multivariate analysis revealed that two factors, the NLR and prior docetaxel chemotherapy, were associated significantly with CSS and OS in CRPC patients who were treated with ENZ. In addition, the NLR might be a useful biomarker for predicting the prognosis of CRPC patients who are treated with ENZ.

## Figures and Tables

**Table 1:** 

		Number (%) or Median(mean ±SD)		
Variables	Total	NLR < 2.14	NLR≧2.14	p value
Number	106	35(33%)	71(67%)	
Age (years)		74 (72.0±1.56)	73 (72.4±0.84)	0.77
Gleason Sum				
<9	46(43.4%)	15(42.8%)	31(43.7%)	1.00
≧9	51(48.1%)	17(48.6)	34(47.9%)	
Unknown	9(8.5%)	3(8.6%)	6(8.4%)	
Metastasis	73(68.8%)	22(62.8%)	51(71.8%)	0.598
Visceral metastases	9(8.8%)	4(11.4%)	5(7.0%)	0.474
Performance status				
0	51	17	34	0.611
1	12	4	8	
2	2	1	1	
3	0	0	0	
4	1	1	0	
Unknown	40	12	28	
Pre docetaxel	38	9	29	0.139

**Table 2:** Multivariate analysis for cancer-specific survival.

	HR	95%CI lower	95%CI upper	p value
Gleason Sum ≥9	0.50	0.19	1.32	0.164
Performance Status	1.12	0.52	2.41	0.756
Visceral metastasis	0.53	0.11	2.51	0.428
Pre docetaxel chemotherapy	4.80	1.54	14.98	0.006
NLR≥2.14	8.26	1.59	42.76	0.011

**Table 3:** Multivariate analysis for overall survival.

	HR	95%CI lower	95%CI upper	p value
Gleason Sum ≥9	0.54	0.22	1.34	0.185
Performance Status	1.40	0.75	2.63	0.284
Visceral metastasis	0.51	0.11	2.36	0.395
Pre docetaxel chemotherapy	3.48	1.30	9.35	0.013
NLR≥2.14	4.57	1.31	15.96	0.017

## Data Availability

The data used to support the findings of this study are available from the corresponding author upon request.
